# Sir George Newman's Report

**Published:** 1919-01-04

**Authors:** 


### THE MEDICAL WORK OF THE BOARD OF EDUCATION.

### Sir George Newman's Report.

For the future which lies before us, we want not only more children but better children, as the foundation of a new people! This, the closing sentence of Sir George Newman's foreword to his tenth annual report, is the most fitting introduction to it. It is at once the justification of the medical work of the Board and a test by which to prove it.

Striking figures show the size of the task, how much has been done—despite the upset of the war—and how much remains to do. During the year 1917, over five million children attended the Public Elementary Schools; the School Medical Officers examined 1,362,063 of them and found that 45.7 per cent. of this number showed some defect; 33.4 per cent. of those examined needed treatment, and of these 66.3 per cent. got it; at least 50 per cent. of the children need treatment for their teeth but in only 151 educational areas out of a total of 318 has any provision been made; special schools for blind, deaf, crippled, and feeble-minded, epileptic and tuberculous children have been set up to about one per cent. of the total number of school children, but this is enough for less than one half of those who need them. How many-sided is the work of the doctors of the Board and how closely it is bound up with the system of education are shown by this list of the chief matters touched upon in the report—the medical inspection of school children; the organisation of treatment, including the special arrangements made for diseases of the ear, throat, nose, eyes, teeth and skin; the work of following-up defective children; the remedial measures for uncleanness; the provision of special and open-air schools, and of schools for mothers and of day-nurseries; the problem of the mentally subnormal and epileptic child; the sanitation of school buildings and the prevention of epidemics; the teaching of hygiene and mother-craft, and of physical exercises, games, swimming, and dancing; the provision of school meals and of school and holiday camps; and the control of juvenile employment. When one reads this list, remembers that 1917 was only the tenth year of the work of the School Medical Service, and that the past three years have taken a heavy toll of its doctors and nurses, one wonders that so much has been done rather than that so much has been left undone.

Sir George Newman insists that the School Medical Service forms part of the system of preventive medicine. For a large part of its work the statement is undoubtedly true. But another large part is not preventive, but is a very valuable index of the failure of our preventive methods. The part of the report dealing with the defects found amongst school children is a terrible indictment of our public health system. A surprise examination was made of two sets of 300 unselected older children each in typical London and country schools. The results were disheartening; 8.5 per cent. of the children were absent from school on grounds of more or less chronic ill health; only 40 per cent. were well nourished, only 70 per cent. had clean heads and 73 per cent. clean bodies; 49 per cent. had carious teeth; 11 per cent. suffered from disease of nose and throat; 24 per cent. had defective eyesight; 15 per cent. were more or less deaf; 6 per cent. showed severe anæmia; and of middle-ear disease, of organic heart disease, of skin disease, and of pronounced spinal curvature there were 4 per cent. in each class. Summing up the results of this inquiry Dr. C. J. Thomas says: "Thus a little more than five per cent. of the children seen were suffering from defects most of which might be prevented or alleviated if dealt with betimes, defects that interfere with their profiting to a due and reasonable extent from the education provided, and will prevent them from playing their fair and proper part as citizens."

School inspection cannot prevent these defects. The teaching of mother-craft in the schools and the training of mothers and expectant mothers in the Infant Welfare Centres may be expected to have a good effect, but of greater importance is the provision of healthy homes and workshops, of good wages, of fewer hours of work and of efficient medical treatment and guidance in the years before school life begins. And one may ask why is the teaching of the care of children given to the girls only. If the teaching were given to both the boys and girls there would be, as there ought to be, a double guard upon the health of the child. But more is needed. The older boys and girls should be taught the requirements of healthy parentage. Then there would be hope that the race might escape some of the avoidable hereditary evils that now fall upon it. It is indeed of more importance that the sense of the obligations of the parent to the child should be quickened in the man than in the woman. With the latter it is innate and born of her physical life: with the former it is born of centuries of education and of the ordered life of the community.

The report deals very fully with the effects of the war upon the health of the child. It is satisfactory to find that, speaking generally, the children are better clothed, better shod, better fed, and in better health than they were before the war. The explanation given is that the parents are better off, and can buy more food under present conditions than they could in times when food supplies were greater and wages lower. There is no evidence that illness has been caused by the eating of war-bread, but there has been a serious increase in the adulteration of milk. Unfortunately there has been also an increase in uncleanliness and in such conditions as ringworm, scabies, and conjunctivitis, partly due to the lessening of parental supervision and partly to the introduction of infection from returning troops. Contrary to expectations, there is no evidence of any increase in the incidence of

**Medical Work of the Board of Education**—(*cont.*).

nervous complaints amongst school children as the result of air raids. The fears expressed when the Daylight Saving Act was passed have, however, proved to be well grounded. There is evidence to show that one effect has been to deprive children of an hour of sleep daily and that their health and education suffer in consequence.

The report shows that increasing attention is being paid to physical education. One most satisfactory feature is that the Board of Education has learnt that games are more important than formal drill and gymnastics. The war has had an unexpected influence here. The organisation of games behind the lines has taught many men for the first time in their lives the joy of play. As the report points out, they will come home wishful to go on playing, unsatisfied to be lookers-on at games and resolved that their children shall be taught to play. So we may look forward to the more general teaching of games, swimming, and dancing, and to a time when every school will have its playing grounds and camping site as a matter of course. The scenes in our streets during the week following the armistice should have driven home to all of us the pressing need of this. Here was a people who knew not now to show its joy and relief except in meaningless noise and turmoil. It could not sing, its dancing would have shamed a Bushman, its manifestations of joy were on a level of or infinitely below that of a corroboree. Its very elect could only give it a firework display banal in conception and puerile in execution. In the times that are to be, such a day may call forth the guilds with their bands and banners and organised marching and may find the crowd able to join in folk dances and in the concerted singing of worthy national songs.

In this short review it is impossible to touch upon all the points of interest and importance in the Report. Any doctor, anyone interested in the welfare of the nation, will find the time spent in reading it amply repaid. He will find the facts put forth tensely and vividly and he will close the book glad that so much has been done and full of hope for the future.

